# Body mass index relates weight to height differently in women and older adults: serial cross-sectional surveys in England (1992–2011)

**DOI:** 10.1093/pubmed/fdv067

**Published:** 2016-10-17

**Authors:** Matthew Sperrin, Alan D. Marshall, Vanessa Higgins, Andrew G. Renehan, Iain E. Buchan

**Affiliations:** 1 Health eResearch Centre, Farr Institute, University of Manchester, Manchester M13 9PL, UK; 2 Cathie Marsh Institute for Social Research, School of Social Sciences, University of Manchester, Manchester, UK; 3 Institute of Cancer Sciences, University of Manchester, Manchester, UK

**Keywords:** adiposity, body height, body mass index, body weight, health survey for England, stature, weight for height

## Abstract

**Background:**

Body mass index (BMI) tends to be higher among shorter adults, especially women. The dependence of BMI–height correlation on age and calendar time may inform us about temporal determinants of BMI.

**Methods:**

Series of cross-sectional surveys: Health Survey for England, 1992–2011. We study the Benn Index, which is the coefficient in a regression of log(weight) on log(height). This is adjusted for age, gender and calendar time, allowing for non-linear terms and interactions.

**Results:**

By height quartile, mean BMI decreased with increasing height, more so in women than in men (*P* < 0.001). The decrease in mean BMI in the tallest compared with the shortest height quartile was 0.77 in men (95% CI 0.69, 0.86) and 1.98 in women (95% CI 1.89, 2.08). Regression analysis of log(weight) on log(height) revealed that the inverse association between BMI and height was more pronounced in older adults and stronger in women than in men, with little change over calendar time.

**Conclusions:**

Unlike early childhood, where taller children tend to have higher BMI, adults, especially women and older people, show an inverse BMI–height association. BMI is a heterogeneous measure of weight-for-height; height may be an important and complex determinant of BMI trajectory over the life course.

## Introduction

Body mass index (BMI) relates weight to height in a normalized index that was first published in Quetelet's 18th Century treatise on ‘the average man’.^1^ BMI is defined as weight (in kilograms) divided by height (in metres) squared. As an individual's height and weight can be readily and inexpensively measured, BMI has become a popular heuristic approximation for body fatness in epidemiology and clinical practice. The World Health Organization defined BMI-based fatness categories of underweight (BMI < 18.5 kg/m^2^), normal weight (18.5–24.9 kg/m^2^), overweight (25.0–29.9 kg/m^2^) and obese (≥30.0 kg/m^2^).^2^ Excess body weight (overweight and obese) is a major risk factor for mortality and morbidity from cardiovascular disease,^3^ type 2 diabetes^4^ and incident cancer,^5,6^ causing 3 million deaths each year worldwide.^7^ National surveys show that adiposity, as measured by BMI, has increased over the past decades in many populations across the world.^8^ However, the rising trends have slowed down since approximately 2000 in England^9^ and other countries.^8,10^

BMI is not a measure of body fat amount or distribution. Much debate has argued that other anthropometric measures, such as waist circumference and waist-to-hip ratio, are better predictors of disease outcome compared with BMI,^11,12^ though recent meta-analyses suggest that these measures are no more informative than BMI for cardiovascular disease^3^ or type 2 diabetes.^4^ In adult population studies, there is little debate about the relationship of weight to height, but this should be questioned where height is a risk factor for disease, for example, in breast cancer among women.^13^ When using BMI, it is commonly assumed that:^14^ (i) BMI is strongly correlated with weight, but independent of height; and (ii) BMI correctly captures the relationship between weight and height. This may not be true, particularly at earlier points in the life course. We showed that BMI had been rising more in the taller (faster growing) 3 year olds than in their shorter peers, suggesting a causal drive to increasing adiposity in young children that involves both growth and appetite.^15^ Others showed similar non-independence of height and BMI among 7–12 year olds.^16^

In 1971, Benn described BMI's fundamental statistical relationship as (Weight/Height P) where the height power *P* (termed the *Benn Index*) is estimated by log–log regression.^17^ Specific examples for the value of the Benn Index include the Ponderal Index (or Rohrer Index)^18^ where *P* = 3, the Human Body Shape Index where *P* = 2.8,^19^ and the standard BMI calculation or Quetelet index where *P* = 2. Many studies in the paediatric literature have shown that the value of the Benn Index varies with gestational age^20^ and during childhood development.^21–23^ Research in adults suggests that BMI is negatively correlated with height, especially in women.^14^ In this study, we seek to extend previous research in two ways. First, we examine the impact of age on the BMI–height relationship between ages 16 and 75 years. Second, recognizing the increasing prevalence of overweight and obesity in England over the past two decades,^24^ we explore whether or not the BMI–height relationship has changed over this time period. Methodologically, we extend to an adult population our analyses of the BMI–height relationship in young children^15^ and determine whether this relationship differs by gender, by age group, and over time, and explore for influences of potential confounders, such as smoking and income.

## Methods and procedures

### Data source

The Health Survey for England (HSE) is a series of annual cross-sectional surveys, piloted in 1991 and run in full since 1992, to monitor the health of the English population. The survey methodology follows a multistage, stratified probability sampling design and originally had a sample of 4000, but this was increased to around 16 000 in 1994. A fresh sample of participants is invited each year by selecting private households at random in a geographically dispersed sampling frame. Socio-demographic information and height/weight measurements were collected using standardized procedures by trained interviewers at the homes of participants. Weight is measured to the nearest 100 g using electronic scales after removal of shoes or bulky clothing (participants were not weighed if they were pregnant, unsteady on their feet or chair-bound). Height, to the nearest millimetre, was measured using a portable stadiometer. Previous surveys reported on average 70% of households agreed to an interview, and BMI was available from around 90% of those interviewed—with some variation by year and region.^25,26^

We have previously considered missingness of height/weight/BMI values in HSE and shown that there is slightly more missingness in later survey years, but there are no discernible differences between missing and non-missing cases for age, socioeconomic status, educational status, smoking status and income classes, i.e. it is robust to assume that BMI values are missing completely at random within gender.^9^

### Data capture and inclusion criteria

We created a Health Survey for England dataset from 1992 to 2011, available for download from http://ukdataservice.ac.uk. We included only individuals where both a valid height and weight measurement were recorded. We restricted the sample to represent an adult population that we defined as age ≥16 and <75 years. Those who were considered by the interviewer to have unreliable measurements were excluded from the analysis. We excluded ‘boost samples’ and did not apply survey weights, as these were only introduced in 2003, and the HSE is generally believed to be representative without applying these weights. We extracted the core variables of person identifier, year, age and validated (with height and weight) BMI, together with smoking status and equivalized household income for sensitivity analysis.

### Statistical analyses

The relationship between height group (by quartile) and BMI was first explored graphically by gender and calendar time; simple regressions were used to assess the dependence of BMI on height, stratified by gender. An F test was used to test for an interaction between height quartile and gender in a regression model with BMI as the outcome and height quartile, gender, and their interaction as predictors.

We then used a more complex regression model to investigate interactions and non-linear effects, in which log(weight) was taken as the response and regressed on log(height) as a continuous variable. We fitted separate models for males and females; we corrected for age and calendar time and allowed interactions between them. The variable log(height) was constrained to be linear, but age and calendar time were allowed to have non-linear effects. The key output was the log(height) coefficient—the Benn Index—which can vary according to the other covariates through its interactions.

The model fitting procedure was as follows. For each gender, we first fitted a model constrained to be linear in log(height), with fractional polynomial terms^27^ for age, calendar time and the interaction between age and calendar time. The choice of fractional polynomial terms was made using the iterative procedure of Sauerbrei and Royston.^28^ We then considered potential interaction terms between log(height) and each selected fractional polynomial term for inclusion in the model, using forward selection. Analyses were carried out using *R*,^29^ with fractional polynomials fitted using the *mfp* package.^30^

## Results

### Exploratory analyses

Table 1 shows demographic characteristics for each year of the HSE (1992–2011). As previously reported, we observed that the mean BMI and mean age increased per survey year in men and women, with this increase slowing down after 2001.^9^ Mean height increased per survey year in men (0.044 cm per year, *P* < 0.001) and in women (0.033 cm per year, *P* < 0.001); see Supplementary data, Figure S1. The correlation between height and BMI is *ρ* = −0.07 in men and *ρ* = −0.14 in women; more details of correlations between height, weight and BMI over time are given in Supplementary data, Table S1.

**Table 1:** Demographics in the Health Survey for England dataset, after restricting to individuals with valid BMI measurements. Where appropriate these are given in the form ‘mean (standard deviation)’

*Year*	*Men*						*Women*			
	*Sample size*	*% male*	*BMI*	*Weight (kg)*	*Height (cm)*	*Age*	*BMI*	*Weight (kg)*	*Height (cm)*	*Age*
1992	6200	48	25.7 (4.0)	78.7 (13.3)	174.9 (7.2)	42.9 (16.4)	25.4 (5.1)	66.3 (13.7)	161.6 (6.6)	43.2 (16.5)
1993	14 333	48	25.9 (3.9)	79.3 (13.0)	174.7 (7.1)	43.1 (16.2)	25.7 (4.9)	67.1 (13.0)	161.6 (6.6)	43.6 (16.3)
1994	13 706	47	26.0 (3.9)	79.6 (13.2)	175.0 (7.2)	43.0 (16.3)	25.8 (5.0)	67.3 (13.4)	161.6 (6.7)	43.7 (16.5)
1995	13 564	47	26.1 (3.9)	79.9 (13.1)	174.8 (7.0)	43.6 (16.2)	25.9 (5.0)	67.3 (13.1)	161.3 (6.4)	43.6 (16.3)
1996	14 089	47	26.3 (4.0)	80.4 (13.5)	174.7 (7.1)	43.6 (16.1)	26.0 (4.9)	67.6 (13.1)	161.4 (6.5)	43.7 (16.2)
1997	7466	47	26.5 (4.2)	81.0 (13.9)	174.8 (7.0)	43.4 (15.9)	26.2 (5.4)	68.2 (14.2)	161.4 (6.5)	43.6 (15.9)
1998	13 365	47	26.5 (4.2)	81.2 (13.8)	174.8 (7.1)	43.6 (16.0)	26.4 (5.3)	68.8 (14.1)	161.5 (6.5)	43.8 (16.0)
1999	6478	47	26.5 (4.3)	81.1 (14.5)	174.8 (7.1)	44.1 (16.0)	26.4 (5.3)	68.6 (14.2)	161.4 (6.6)	43.6 (15.8)
2000	6466	47	26.9 (4.3)	82.1 (14.3)	174.8 (7.1)	43.7 (15.9)	26.5 (5.5)	69.2 (14.5)	161.5 (6.5)	44.4 (15.6)
2001	12 800	46	27.0 (4.4)	82.8 (14.4)	175.0 (7.0)	44.6 (16.0)	26.8 (5.5)	69.8 (14.5)	161.6 (6.5)	44.3 (15.8)
2002	6033	46	26.9 (4.6)	82.6 (15.1)	175.1 (7.0)	43.6 (16.0)	26.7 (5.5)	69.8 (14.6)	161.7 (6.5)	44.3 (16.0)
2003	12 170	46	27.1 (4.5)	83.2 (14.9)	175.0 (7.0)	45.0 (16.0)	26.8 (5.6)	70.0 (14.9)	161.6 (6.5)	44.9 (15.9)
2004	5162	44	27.3 (4.5)	83.8 (15.2)	175.1 (7.2)	45.7 (16.1)	26.9 (5.5)	70.4 (14.7)	161.8 (6.5)	46.0 (15.7)
2005	5910	46	27.1 (4.6)	83.6 (15.1)	175.4 (7.2)	44.8 (16.1)	27.0 (5.7)	70.7 (15.1)	161.8 (6.5)	44.7 (15.6)
2006	11 139	46	27.5 (4.6)	84.4 (15.3)	175.2 (7.2)	46.1 (16.0)	27.0 (5.6)	70.6 (15.0)	161.9 (6.6)	45.5 (15.8)
2007	5477	46	27.3 (4.8)	84.1 (15.7)	175.4 (7.2)	45.7 (16.2)	26.9 (5.4)	70.4 (14.4)	161.8 (6.5)	45.7 (15.8)
2008	11 814	46	27.4 (4.7)	84.3 (15.5)	175.4 (7.2)	45.5 (16.2)	27.0 (5.7)	70.8 (15.1)	161.9 (6.5)	45.4 (16.1)
2009	3654	47	27.3 (4.7)	84.2 (15.3)	175.5 (7.2)	46.4 (16.5)	27.2 (5.9)	71.2 (15.3)	161.9 (6.6)	45.6 (16.2)
2010	6378	45	27.7 (4.8)	85.5 (16.1)	175.4 (7.0)	46.4 (16.3)	27.4 (6.0)	71.9 (15.9)	162.2 (6.7)	45.7 (15.7)
2011	6436	45	27.5 (4.8)	84.7 (15.9)	175.6 (7.2)	46.4 (16.0)	27.2 (5.8)	71.6 (15.7)	162.2 (6.6)	45.8 (16.0)
Total	182 640	47	26.7 (4.4)	81.9 (14.5)	175.0 (7.1)	44.4 (16.2)	26.5 (5.4)	69.1 (14.4)	161.6 (6.5)	44.4 (16.1)

Source: Authors' own calculations based on the Health Survey for England (1992–2011).

### BMI trends over time by height group (in quartiles)

**Fig. 1:**
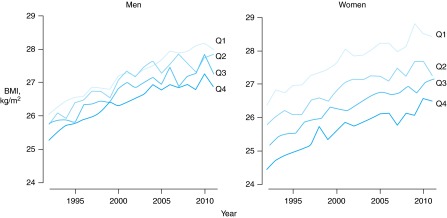
English mean BMI by height quartile from 1992 to 2011. Left: men; right: women. Q1 to Q4 as Quartile 1 (shortest) to Quartile 4 (tallest).

Figure 1 shows the rise in BMI that occurred between 1992 and 2011 for both men and women, stratified by height quartile. Women's height groups have a much larger and more consistent separation of their BMI trajectories than men. Tall women had lower BMI, on average, compared with short women. For the tallest quartile of men compared with the shortest quartile, mean BMI was 0.77 kg/m^2^ (95% CI [0.69, 0.86], *P* < 0.001) lower; for the taller women, the corresponding difference in mean BMI was more than two and a half times larger at 1.98 kg/m^2^ (95% CI [1.89, 2.08], *P* < 0.001); results for all of the height groups are detailed in Table 2. The F test on the combined regression model demonstrated that the interaction between gender and height quartile was statistically highly significant (*P* < 0.001). Sensitivity analyses of these BMI differences stratified by smoking status and income are shown in Supplementary data, Table S2.

**Table 2:** Reduction in mean BMI by height quartile, stratified by gender (Quartile 1 is the baseline, and the mean BMI is presented for this category)

*Height quartile*	*Point estimate (95% CI) for reduction in mean BMI (kg/m^2^)*	
	*Men*	*Women*
1 (shortest)	Baseline: 27.09 (27.03, 27.15)	Baseline: 27.50 (27.43, 27.57)
2	−0.18 (−0.26, −0.10)	−0.83 (−0.92, −0.73)
3	−0.47 (−0.55, −0.38)	−1.33 (−1.42, −1.23)
4 (tallest)	−0.77 (−0.86, −0.69)	−1.98 (−2.08, −1.89)

Source: Authors' own calculations based on the Health Survey for England (1992–2011).

### Trends in Benn Index

**Fig. 2:**
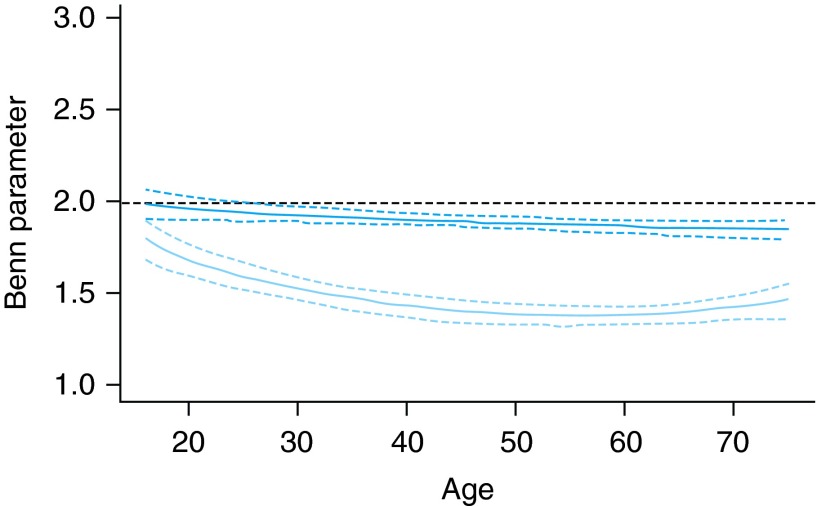
Benn parameter (relative change in predicted weight when height is increased by 1%) for various ages. Top line (dark blue), males; bottom line (light blue), females. Solid line, expected change; dashed line, 95% confidence limits. Interpretation: a Benn Index <2 implies taller people tend to have lower BMI.

We then fit the log(weight) against log(height) regression models. Technical details of final fitted models are given in Supplementary data, Tables S3 and S4. We examined the weight–height relationships over age and gender (Fig. 2)—setting the year to 2011. The Benn Index was slightly <2 in men, and much <2 in women. This suggests a stronger gradient between BMI and height in women (taller women tending to have lower BMI). In men, the Benn Index decreased further from 2 with increasing age (linear trend, *P* = 0.005), suggesting a strengthening of the above relationship with age. In women, the Benn Index followed a quadratic relationship with age (*P* < 0.001), with the relationship strongest around middle age.

**Fig. 3:**
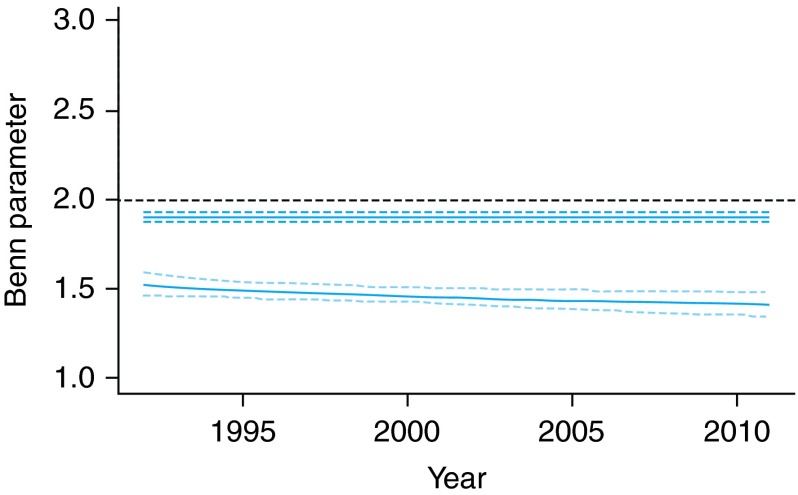
Benn parameter (relative change in predicted weight when height is increased by 1%) over calendar time. Top line (dark blue), males; bottom line (light blue), females. Solid line, expected change; dashed line, 95% confidence limits. Interpretation: a Benn Index <2 implies taller people tend to have lower BMI.

We then explored period effects of the weight–height relationships (Fig. 3)—setting age to 45. There is no change over calendar time in the male Benn Index (the term for this is selected out of the model). There is a slight decrease of the Benn Index over time for women; however, a test of linear trend is not significant (*P* = 0.22).

### Sensitivity analyses

We performed sensitivity analyses taking account of the potential influences of smoking and income status (Supplementary data, Figures S2 and S3). Patterns were generally similar, although Benn indices were slightly larger in current smokers, particularly in men. There was little effect of income status on Benn indices.

## Discussion

### Main finding of this study

Height is inversely associated with BMI in adults. This relationship is larger in women and has generally increased with age. We established this with data from the Health Surveys for England (1992–2011) and analysis of the relationship between BMI and height for adults (aged 16–75) using regressions with log(weight) as the response and log(height) as the predictor, with age, gender and calendar time as additional predictors, allowing for interactions and non-linear terms, and exploring the influence of smoking and other potential confounders.

### What is already known on this topic

BMI is a popular approximation of body fatness, purporting to correct for the relationship between weight and height. However, in pre-pubertal children, there is a positive association between BMI and height (taller = higher BMI),^15,16^ while in adults, there is a negative association between BMI and height, particularly in women.^14^ The relationship between BMI and height has been studied in detail in children;^20–23^ for adults, it is not clear how the BMI–height association relates to age, or whether it has changed over time.

### What this study adds

The excess of weight-for-height among shorter people is negligible in early adulthood (16–20 years), particularly in men, then increases with age. This suggests an inversion of the BMI–height relationship around puberty. There is little evidence of this changing over calendar time, despite the growing levels of obesity.

Examining changes over time in BMI at the population level conflates not only the constituent fat and lean mass factors but also the dynamics of linear growth, stature, adiposity and skeletal musculature. To understand the implications of our findings, the time trends in linear growth and stature must be considered alongside time trends in adiposity, with a life-course perspective.

In early life, greater maternal pregnancy weight gain,^31^ higher birth weight^32^ and faster growth (as measured by taller stature)^15,16^ are associated with higher BMI before puberty. Then higher BMI is associated with earlier onset and completion of puberty and an impaired height gain during puberty.^33,34^ One explanatory mechanism is higher adiposity driving skeletal maturation through its effects on oestrogen metabolism, which applies to boys^35^ as well as girls. However, there is a divergence between the sexes around puberty in their linear growth and overall development in relation to adiposity.^36,37^ The eventual effects of pubertal overweight and obesity on adult attained height are inconsistent in boys, but in girls there appears to be an inversion of the BMI–height association.^38^ So, at the population level, successive cohorts experiencing an increasingly obesogenic environment might show: (i) increasing sexual dimorphism in the BMI–height association and (ii) increasing average attained height if rising determinants of early growth override the accelerated skeletal maturation of high adiposity. Our results are consistent with this explanation. There is, however, a complex nexus of different causal pathways to consider, which is beyond the scope of this research.

In clinical terms, our findings add further caution to the use of BMI as a proxy for adiposity, as it has different meanings between adults of different statures and between the sexes, with an underlying dynamic that most likely applies across the life course.

In public health terms, we have shown that the comparison of weight-for-height between heterogeneous groups, and over time, may require more detailed statistical modelling than simple mean BMI contrasts. Subject to further research, there may be a need for more targeting/adaptation of healthy weight promotion by sex and stature.

Population summaries of BMI represent a heterogeneous mix of weight-for-height relationships that cannot be reduced to Quetelet's kg/m^2^. The bases of these relationships may vary across the life course and by sex. Exploring the nexus of potential causality in the BMI–height associations may reveal some useful targets for tackling obesity.

Based on the findings here and our previous findings in young children,^15,39^ we hypothesize that the BMI trajectory is dependent on height, with taller individuals gaining more weight in childhood and shorter individuals accumulating more weight throughout the life course. This will need to be tested in cohorts with height and weight measured from childhood to old age, ideally at different phases of calendar time with a variety of obesogenic environments acting across the life course. There is a need for more collective, cross-cohort research in this regard.

### Limitations of this study

A limitation of the study is that the data are cross-sectional only; therefore, we cannot study how BMI changes within individuals. In particular, we cannot easily distinguish empirically from our data whether differences in the BMI and height relationship by age are a result of age, period or cohort. We are also basing our analysis on subsamples where BMI measures were available; this may introduce bias if BMI measures are not missing at random; however, we have previously proved this to be unlikely with data from this survey series.^9^

From serial cross-sectional health surveys, we cannot determine whether the findings in this work and elsewhere represent evidence of a causal relationship between height and adiposity, or simply reflect the heterogeneity of BMI as an adiposity measure (across populations, the life course and calendar time). In addition, the direction of possible causality in BMI–height relations may not be the same across the life course.

However, a strength is the use of a large annual survey with comparable sampling and measurement of the English general population over the past two decades.^26^ We have also used refined statistical modelling techniques to examine the changing relationships between height and BMI according to age, gender and calendar time.

## Conclusion

BMI does not reflect the same adjustment of weight to height between the sexes or across age groups. This heterogeneity of BMI must be considered in public health research and surveillance. Longitudinal studies across a variety of cohort samples, populations and environments are needed to investigate whether or not BMI has a meaningful life-course trajectory.

## Supplementary data

Supplementary data are available at the *PUBMED* online.

## Funding

This study was partly supported by the University of Manchester's Health eResearch Centre (HeRC) funded by the Medical Research Council (MRC) Grant MR/K006665/1 and partly funded by the ESRC Obesity eLab Grant (RES-149-25-1076).

## Supplementary Material

Supplementary Data
