# Supplementary material for: Body mass index relates weight to height differently in women and older adults: serial cross-sectional surveys in England (1992–2011)
Source: J Public Health (Oxf). 2016 Oct 17;38(3):607–13. doi: 10.1093/pubmed/fdv067 (PMC5072155; doi:10.1093/pubmed/fdv067)
Supplement: Supplementary Data [file supp_38_3_607__index.html]

Body mass index relates weight to height differently in women and older adults: serial cross-sectional surveys in England (1992–2011) — Body mass index relates weight to height differently in women and older adults: serial cross-sectional surveys in England (1992–2011) — Body mass index relates weight to height differently in women and older adults: serial cross-sectional surveys in England (1992–2011) — Supplementary Data 

# Body mass index relates weight to height differently in women and older adults: serial cross-sectional surveys in England (1992–2011)

## Supplementary Data

Supplementary Data

- Supplementary Data - Docx file
