# Supplementary material for: Body mass index relates weight to height differently in women and older adults: serial cross-sectional surveys in England (1992–2011)
Source: J Public Health (Oxf). 2016 Oct 17;38(3):607–13. doi: 10.1093/pubmed/fdv067 (PMC5072155; doi:10.1093/pubmed/fdv067)
Supplement: Supplementary Data [file supp_fdv067_fdv067supp.docx]

**Electronic supplementary material**

Body mass index relates weight to height differently in women and older adults; serial cross-sectional surveys in England (1992-2011)

Matthew Sperrin, Alan D Marshall, Vanessa Higgins, Andrew G Renehan, Iain E Buchan

Correspondence to:

Dr Matthew Sperrin

Health eResearch Centre, Farr Institute

University of Manchester

Manchester M13 9PL

United Kingdom

Tel: +44 (0)161 306 7629

E-mail: matthew.sperrin@manchester.ac.uk

**Supplementary Fig. 1:** Mean height by gender from 1992 to 2011.

Source: Authors’ own calculations based on the Health Survey for England (1992-2011)

**Supplementary Table 1**: Correlations between height and weight, and between height and BMI, in men and women, over calendar time.

|  | Men | | Women | |
| --- | --- | --- | --- | --- |
|  | Correlation between height and weight | Correlation between height and BMI | Correlation between height and weight | Correlation between height and BMI |
| 1992 | 0.42 | -0.07 | 0.26 | -0.14 |
| 1993 | 0.43 | -0.08 | 0.26 | -0.16 |
| 1994 | 0.43 | -0.07 | 0.27 | -0.15 |
| 1995 | 0.41 | -0.08 | 0.26 | -0.15 |
| 1996 | 0.41 | -0.07 | 0.27 | -0.15 |
| 1997 | 0.39 | -0.08 | 0.24 | -0.15 |
| 1998 | 0.40 | -0.08 | 0.27 | -0.12 |
| 1999 | 0.41 | -0.04 | 0.27 | -0.13 |
| 2000 | 0.39 | -0.08 | 0.24 | -0.14 |
| 2001 | 0.38 | -0.08 | 0.24 | -0.15 |
| 2002 | 0.37 | -0.07 | 0.23 | -0.15 |
| 2003 | 0.38 | -0.07 | 0.24 | -0.14 |
| 2004 | 0.40 | -0.05 | 0.24 | -0.15 |
| 2005 | 0.38 | -0.08 | 0.22 | -0.15 |
| 2006 | 0.37 | -0.08 | 0.25 | -0.14 |
| 2007 | 0.36 | -0.09 | 0.24 | -0.15 |
| 2008 | 0.37 | -0.08 | 0.24 | -0.14 |
| 2009 | 0.36 | -0.10 | 0.19 | -0.19 |
| 2010 | 0.37 | -0.06 | 0.24 | -0.15 |
| 2011 | 0.36 | -0.08 | 0.26 | -0.12 |
| Overall | 0.39 | -0.07 | 0.25 | -0.14 |

**Supplementary Table 2:** Reduction in mean BMI in 4^th^ height quartile compared with 1^st^, stratified by

gender, smoking status and household income.

|  | **Point estimate (95% CI) for reduction in mean BMI (kg/m^2^)** | |
| --- | --- | --- |
| **Strata** | **Men** | **Women** |
| Current smoker | -0.13 (-0.29,0.02) | -1.09 (-1.28,-0.91) |
| Ex smoker | -0.89 (-1.04,-0.74) | -2.17 (-2.38,-1.96) |
| Never smoker | -0.90 (-1.03,-0.77) | -2.34 (-2.47,-2.21) |
| Top income | -0.82 (-1.05,-0.59) | -1.73 (-2.01,-1.45) |
| Upper middle income | -0.76 (-1.01,-0.51) | -1.34 (-1.64,-1.05) |
| Middle income | -0.97 (-1.25,-0.70) | -1.81 (-2.12,-1.51) |
| Lower middle income | -1.03 (-1.35,-0.71) | -2.07 (-2.40,-1.74) |
| Lowest income | -1.09 (-1.46,-0.72) | -1.81 (-2.18,-1.43) |

**Final Fitted Models**

Fits for the final models are presented below. These are technical details of the final fractional polynomial models. For example, the male Benn index model (**Supplementary Table 3**) implies the model for the expected log(weight):

E[log(weight)] = -6.31 + 2.00*log(height) + 0.33*((mage + 29.5)/10)^0.5 – 0.30*((mage + 29.5)/10)
+ 0.04*((myr + 10)/10)^0.5 + 0.04* log(height)*((mage + 29.5)/10).

**Supplementary Table 3**: Male Benn index model: ‘mage’ denotes centred age and ‘myr’ denotes centred calendar year. A colon ‘:’ denotes an interaction term.

|  | **Estimate** | **Std. Error** | **t value** | **Pr(> \|t\|)** |
| --- | --- | --- | --- | --- |
| (Intercept) | -6.31 | 0.26 | -24.04 | 0.00 |
| log(height) | 2.00 | 0.05 | 39.61 | 0.00 |
| ((mage + 29.5)/10)^0.5 | 0.33 | 0.01 | 30.70 | 0.00 |
| ((mage + 29.5)/10) | -0.30 | 0.09 | -3.44 | 0.00 |
| ((myr + 10)/10)^0.5 | 0.04 | 0.00 | 13.51 | 0.00 |
| log(height):((mage + 29.5)/10) | 0.04 | 0.02 | 2.41 | 0.02 |

**Supplementary Table 4**: Female Benn index model: ‘mage’ denotes centred age and ‘myr’ denotes centred calendar year. A colon ‘:’ denotes an interaction term.

|  | **Estimate** | **Std. Error** | **t value** | **Pr(> \|t\|)** |
| --- | --- | --- | --- | --- |
| (Intercept) | -6.31 | 0.40 | -15.96 | 0.00 |
| log(height) | 2.02 | 0.08 | 26.07 | 0.00 |
| ((mage + 29.5)/10)^0.5 | 1.65 | 0.26 | 6.44 | 0.00 |
| ((mage + 29.5)/10)^3 | -0.01 | 0.00 | -3.49 | 0.00 |
| ((myr + 10)/10)^0.5 | 0.52 | 0.24 | 2.18 | 0.03 |
| log(height):((mage + 29.5)/10)^0.5 | -0.30 | 0.05 | -5.98 | 0.00 |
| log(height):((mage + 29.5)/10)^3 | 0.00 | 0.00 | 3.33 | 0.00 |
| log(height):((myr + 10)/10)^0.5 | -0.09 | 0.05 | -1.94 | 0.05 |

**Supplementary Fig. 2**: Benn coefficient over year and age in different smoking strata. Blue - males, pink – females. Solid line – expected change, dashed line – 95% confidence limits.

**Supplementary Fig. 3**: Benn coefficient over year and age in different income quintiles. Blue - males, pink – females. Solid line – expected change, dashed line – 95% confidence limits.
